# Cumulative risk of false positive test in relation to breast symptoms in mammography screening: a historical prospective cohort study

**DOI:** 10.1007/s10549-016-3931-8

**Published:** 2016-08-05

**Authors:** Deependra Singh, Janne Pitkäniemi, Nea Malila, Ahti Anttila

**Affiliations:** 1 Finnish Cancer Registry, Unioninkatu 22, 00130 Helsinki, Finland; 2 School of Health Sciences, University of Tampere, Arvo Building, Lääkärinkatu 1, 33014 Tampere, Finland

**Keywords:** Breast cancer symptoms, False positive, True positive, Mammography, Screening, Lump

## Abstract

Mammography has been found effective as the primary screening test for breast cancer. We estimated the cumulative probability of false positive screening test results with respect to symptom history reported at screen. A historical prospective cohort study was done using individual screening data from 413,611 women aged 50–69 years with 2,627,256 invitations for mammography screening between 1992 and 2012 in Finland. Symptoms (lump, retraction, and secretion) were reported at 56,805 visits, and 48,873 visits resulted in a false positive mammography result. Generalized linear models were used to estimate the probability of at least one false positive test and true positive at screening visits. The estimates were compared among women with and without symptoms history. The estimated cumulative probabilities were 18 and 6 % for false positive and true positive results, respectively. In women with a history of a lump, the cumulative probabilities of false positive test and true positive were 45 and 16 %, respectively, compared to 17 and 5 % with no reported lump. In women with a history of any given symptom, the cumulative probabilities of false positive test and true positive were 38 and 13 %, respectively. Likewise, women with a history of a ‘lump and retraction’ had the cumulative false positive probability of 56 %. The study showed higher cumulative risk of false positive tests and more cancers detected in women who reported symptoms compared to women who did not report symptoms at screen. The risk varies substantially, depending on symptom types and characteristics. Information on breast symptoms influences the balance of absolute benefits and harms of screening.

**Electronic supplementary material:**

The online version of this article (doi:10.1007/s10549-016-3931-8) contains supplementary material, which is available to authorized users.

## Introduction

Organized screening programs for breast cancer have been estimated to reduce breast cancer mortality by about 23 % among those invited. On the other hand, however, it has also been shown to increase the risk of cumulative false positive results by about 20 % [1]. These estimates describe mainly screening programs that invite women aged 50–69 or 50–74 years. There is no clear evidence on effectiveness of systematic clinical breast examination without mammography or of breast self-examination [1, 2]. In addition to sole mammography as the screening test, some programs or trials have performed clinical or physical examination [3]. Clinical examination means systematic palpation by specifically trained staff [3]. However, the clinical examination in Finland is done by collecting self-reported information on symptoms during the screening examination as well as inspection of breasts by the radiographer.

Self-reported symptoms as well as radiographer reports on observations have been a part of the mammography screening program in Finland, since the program started in the late 1980s [3, 4]. Cross-sectional studies have indicated that symptoms have important consequences on the performance of screening [5–7]. There is a risk that harms of screening may increase, as information on symptoms launch further assessments not dealing with breast cancer. The findings of the physical examination may also relate to long-term patterns over several screening rounds.

The main purpose of this study was to estimate the cumulative probability of false positive mammography tests and true positives in women’s visits with symptoms, compared with those visits with no reported symptoms at mammography screening in the Finnish programme in women aged 50–69 years. In addition, we estimated the risk of false positive test and true positive with accumulated same symptom or any symptoms in the screening history.

## Materials and methods

### Study design, setting, and data source

The current study is a register-based cohort study, which utilizes the screening visit history of women who attended the mammography screening program in Finland. The program invited women aged 50–69 years every second year for mammography screening in special organized clinics. Information on breast cancer screening has been registered at the Mass Screening Registry which is part of the Finnish Cancer Registry. The women were asked about breast symptoms at the visit. Any symptoms (lump, retraction, secretion, mole, and scar) women had during the past 2 months were recorded on the mammography form (http://www.cancer.fi/@Bin/44068785/Mammography+form_2006.pdf). The mammography screening examination was two-view for both breasts. The detailed mammography screening process has been described earlier [5]. The registration coverage increased with time, from 51.2 % in 1992 to 90 % in 1998 and virtually 100 % in 2005 and afterwards [4].

The current study population included 413,611 women who were invited for the first time at age 50–51 years in 1992–2004 and were followed up until 2012. Altogether, 2,627,256 invitations were identified during the period 1992–2012, out of which 2,283,706 (87 %) visits were made with an average of 5.5 visits per woman. Records with missing data on symptoms were excluded from the analysis (Table 1). The maximum number of visits per woman was 10, and visits exceeding 10 (145 visits) due to migration within the country were excluded from the current analysis.

### Definition of variables

Test positives are those with primary mammography positive—they are recalled for further assessment (often more mammograms, ultrasound, and needle biopsy) at the screening clinic, if the mammogram indicated any abnormality. The assessment part is called an episode and those with a positive episode are referred to hospital for diagnostics/treatment. Test positives may be episode negative (no referral) or episode positives (referred) and those who are then diagnosed with cancer are true positives at all stages. False positive test are those with negative episode or with a positive episode but no cancer diagnosis at hospital. False positive mammography tests were further classified as at least one or first false positives depending on the screening history: ‘at least one’ if a woman was detected as false positive at any given screening visit irrespective of earlier visit findings and ‘first’ if a woman was detected as false positive at any given screening visit given that mammography in all previous visits was negative. False positive referrals are those with episode positive but no cancer diagnosis in hospital. The average number of visits per woman was defined as the total number of visits made at ages 50–69 years divided by the number of women screened during that period of age. Number of invitations per woman was counted as the number of subsequent invitations a woman received after the first invitation at age 50–51 years.

Women with symptoms reported either by the woman herself or by the radiographer were considered as symptomatic. Symptoms history variable for either lump or retraction or secretion, was created and defined as symptoms reported ever before or at the index visit. Here, index visit means the visit that resulted in a positive test result (either false positive test or true positive test). The possibility of reporting more than one symptom at a single screening visit was also considered. For that, combinations of two symptoms at a time were made as ‘none,’ ‘either’ and ‘both.’ Separate variables for each symptom reported once or more than once in the screening history were created and coded as ‘1 time’ and ‘more than 1 time.’ A separate variable on the absolute number of visits (1–10) per woman was created to compare the probability of false positive test by screening visits, overall versus those with symptoms history.

### Statistical analysis

Lump, retraction, and secretion, the most clinically relevant symptoms, were used for analysis. Let *i* be the index subjects *i* = 1,…, *n* and *j* be the index visits of *i*th subject *j* = 1,…, *J*_*i*_. We note by *P*(*Y*_*ij*_ = 1; *X*_*ij*_) the probability of a false positive test for subject *i* at the *j*th screen given covariates *X*_*ij*_. The cumulative risk of first outcome event after *k* rounds of screening is $q_{k} = 1 - \mathop \prod \nolimits_{j = 1}^{k} \left\{ {1 - P(Y_{ij} = 0;Y_{{i\left( {j - 1} \right)}} = 0, \ldots ,Y_{1} = 0)} \right\}$ [8]. Applying discrete-time hazard model with $logit\left( {P(Y_{ij} )} \right) = X_{'ij}^{'} \beta$ an estimator for cumulative risk can be obtained. A standard logistic regression can be used to get an estimate of the logistic regression model parameters. Suppose that subject *i* had symptoms at the *l*th attended visit. For each subject *i* the visits can be divided into non-symptomatic *j* = 1,…, *l*−1 visits and symptomatic visits *j* = *l*,…, *J* starting from the first symptomatic visit: $\left\{ {\left( {y_{ij} ,X_{ij} = 0} \right);i = 1, \ldots ,I;j = 1, \ldots ,l - 1} \right\}$ and $\left\{ {\left( {y_{ij} ,X_{ij} = 1} \right);i = 1, \ldots ,I;j = l, \ldots ,J} \right\}$. Cumulative risk of false positive test and true positive (cancer diagnosis) was estimated as shown above. Generalized linear regression (GLM) model in R statistical software was used to estimate the effect of an individual symptom as well as combined symptoms on the false positive and true positive probabilities. Confidence intervals at 95 % were estimated using approximate Bayesian inference (INLA) [9].

## Results

**Fig. 1:**
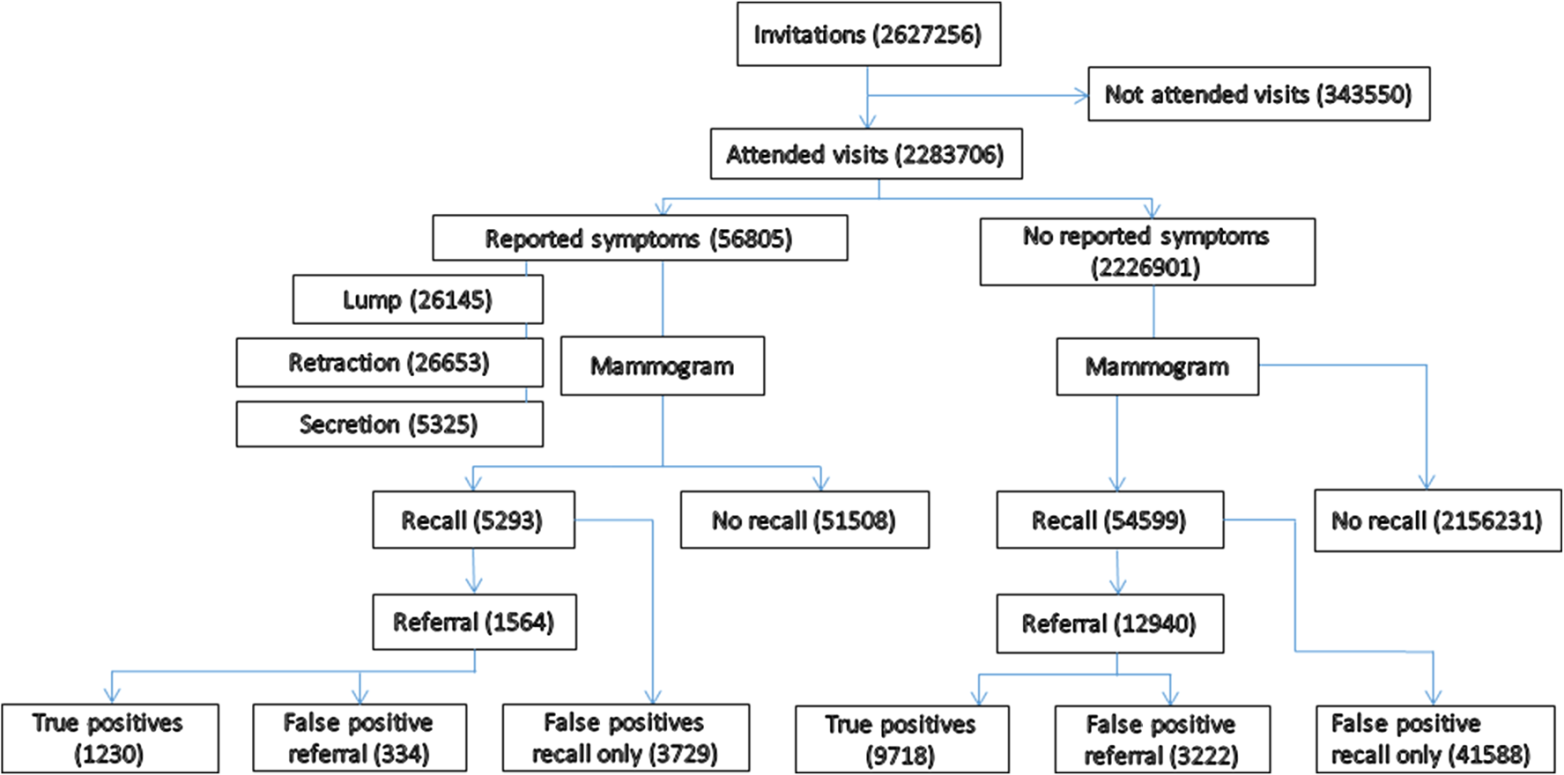
Flow diagram of mammography screening program by symptom status

In 56,805 (2.5 %) visits at least one symptom was reported during the study period in 1992–2012 with a maximum follow-up of 21 years. A lump was reported in 26,145 (1.22 %) visits, retraction in 26,653 (1.59 %) visits, and secretion was reported in 5325 (0.24 %) visits (Fig. 1). There were combined symptoms, as well, with both lump and retraction at 557 visits, lump and secretion at 572 visits, and retraction and secretion at 207 visits. Overall, 48,873 visits (2.1 %) out of total visits had false positive tests. Of these, 44,541 false positive tests were confirmed one time and 4332 false positive test were confirmed more than one time in women screening history. The false positive test percentage at a given visit was 7.2 % (4063 visits) in women with symptoms compared to 2.0 % (44,810 visits) in women with no symptoms. Similarly, the true positive (breast carcinoma) percentage was 2.2 % (1230 visits) in women who reported symptoms compared to 0.4 % (9718 visits) in women with no symptoms (Fig. 1).

**Fig. 2:**
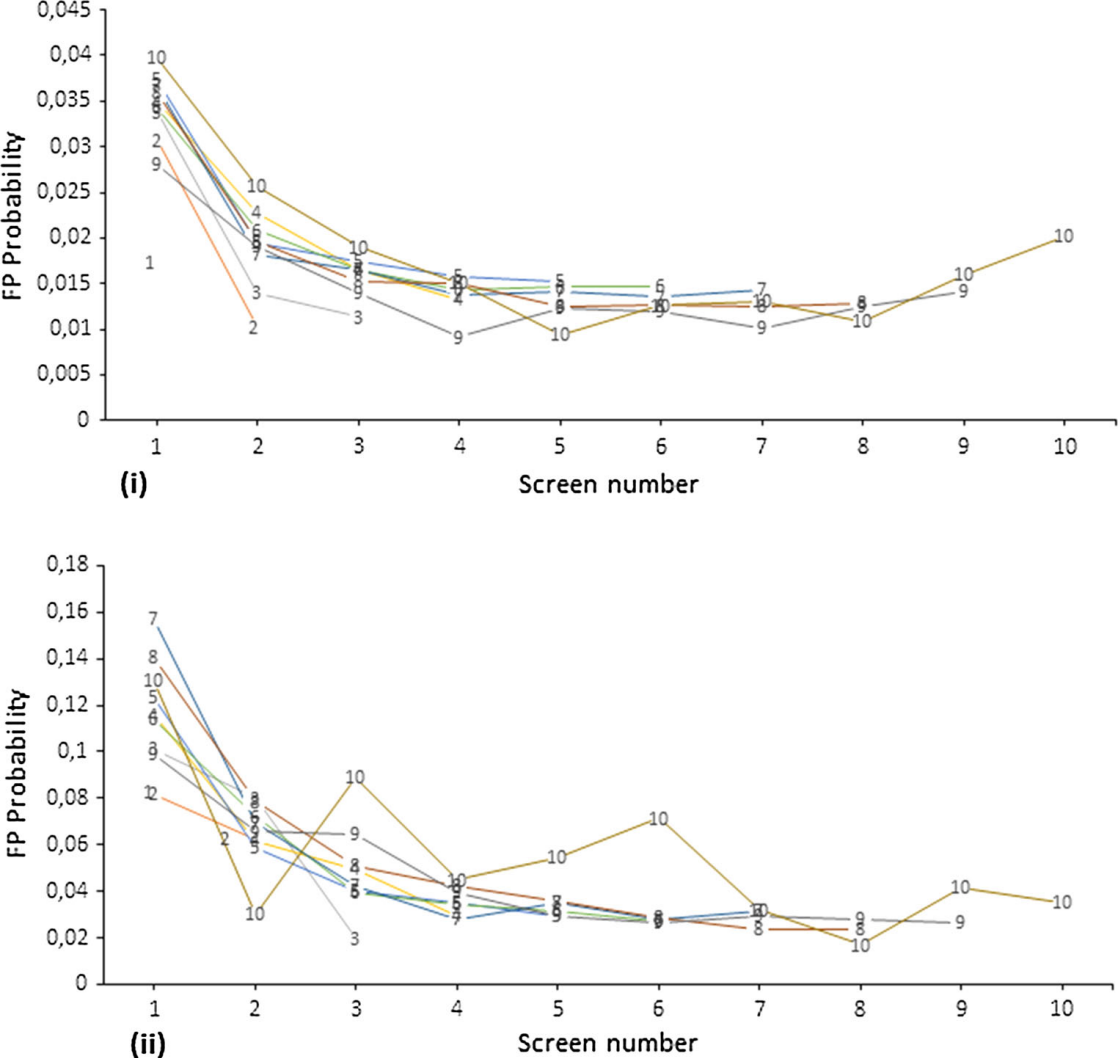
False positive (FP) test probability; overall (**i**) and any symptoms (**ii**), by attended number of screening visits of women

The percentage of women who reported a lump or secretion was higher in younger age groups compared to the older age groups (lump = 1.71 vs. 0.78 %; secretion = 0.32 vs. 0.04 % at 1st and 10th visit, respectively) (Table 1). The false positive proportion among women who reported any symptoms was significantly higher at every visit (order, 1–10) compared to those who did not report any symptoms, overall 7.2 vs. 1.5 %, respectively. False positive test probability based on the absolute number of woman’s visits showed similar difference in women with symptom history compared to women with no history of symptoms (Fig. 2). However, false positive test probability was lower in women who had less (absolute) number of visits compared to those who had completed all possible (ten visits) screening visits. Similarly, the false positive referral and true positive proportions were higher among women who reported symptoms versus no reported symptoms, 2.8 vs. 0.6 % and 2.2 vs. 0.4 %, respectively.

**Table 1:** Number and percentage of symptoms, false positive test and true positives (of symptoms) by number of visits

Symptoms	Number of visit	Number of visits with symptoms (%)	All visits	Missing visits	False positive test		False positive referral		True positives	
					Current symptoms (%)	No current symptoms (%)	Current symptoms (%)	No current symptoms (%)	Current symptoms (%)	No current symptoms (%)
Lump	1	6034 (1.71)	353,348	42,672	922 (15.9)	13,756 (3.98)	69 (1.14)	1196 (0.34)	238 (3.94)	1438 (0.41)
	2	4374 (1.26)	347,915	37,845	569 (13.5)	6890 (2.01)	32 (0.73)	444 (0.13)	146 (3.34)	1110 (0.32)
	3	3814 (1.09)	349,669	24,440	419 (11.4)	6057 (1.76)	33 (0.87)	400 (0.12)	127 (3.33)	1327 (0.38)
	4	3640 (1.08)	337,340	20,857	376 (10.7)	5244 (1.58)	17 (0.47)	317 (0.09)	132 (3.63)	1390 (0.42)
	5	3469 (1.10)	314,846	11,177	378 (11.3)	4564 (1.47)	27 (0.78)	301 (0.10)	118 (3.40)	1536 (0.49)
	6	2442 (1.11)	220,414	3844	270 (11.5)	3047 (1.41)	20 (0.82)	231 (0.11)	97 (3.97)	1233 (0.57)
	7	1522 (1.11)	136,540	2105	126 (8.61)	1868 (1.39)	11 (0.72)	138 (0.10)	59 (3.88)	825 (0.61)
	8	708 (1.07)	66,310	480	62 (9.13)	865 (1.33)	3 (0.42)	64 (0.10)	29 (4.10)	430 (0.66)
	9	122 (1.08)	11,317	36	14 (12.2)	181 (1.63)	0	17 (0.15)	7 (5.74)	79 (0.71)
	10	20 (0.78)	2549	0	4 (21.1)	55 (2.19)	0	6 (0.24)	1 (5.0)	22 (0.87)
	Overall	26,145 (1.22)	2,140,248	143,458	3140 (12.5)	42,527 (1.73)	212 (0.81)	3114 (0.15)	954 (3.65)	9390 (0.44)
Retraction	1	1446 (1.00)	145,113	250,907	81 (5.74)	5529 (3.87)	5 (0.35)	395 (0.27)	36 (2.49)	776 (0.54)
	2	2364 (1.13)	209,617	176,143	69 (2.95)	4271 (2.07)	7 (0.30)	247 (0.12)	27 (1.14)	790 (0.38)
	3	3519 (1.29)	273,512	100,599	109 (3.13)	4772 (1.78)	12 (0.34)	301 (0.11)	31 (0.88)	1169 (0.43)
	4	4597 (1.49)	307,512	50,685	110 (2.42)	4894 (1.62)	8 (0.17)	291 (0.10)	43 (0.94)	1339 (0.44)
	5	5383 (1.76)	306,059	19,964	109 (2.05)	4693 (1.57)	3 (0.06)	313 (0.10)	67 (1.24)	1537 (0.51)
	6	4542 (2.06)	220,381	3877	96 (2.14)	3220 (1.50)	5 (0.11)	246 (0.11)	54 (1.19)	1276 (0.59)
	7	3005 (2.20)	136,540	2105	56 (1.88)	1938 (1.46)	2 (0.07)	147 (0.11)	31 (1.03)	853 (0.64)
	8	1502 (2.27)	66,310	480	30 (2.01)	897 (1.39)	4 (0.27)	63 (0.10)	12 (0.80)	447 (0.69)
	9	244 (2.16)	11,317	36	6 (2.48)	189 (1.72)	0	17 (0.15)	2 (0.82)	84 (0.76)
	10	51 (2.0)	2549	0	2 (3.92)	57 (2.30)	0	6 (0.24)	0	23 (0.92)
	Overall	26,653 (1.59)	1,678,910	604,796	668 (2.54)	30,460 (1.85)	46 (0.17)	2026 (0.12)	303 (1.14)	8294 (0.50)
Secretion	1	1142 (0.32)	358,583	37,437	126 (11.1)	14,557 (4.09)	20 (1.75)	1235 (0.35)	6 (0.53)	1712 (0.48)
	2	1004 (0.28)	360,093	25,667	71 (7.13)	7557 (2.11)	13 (1.29)	483 (0.13)	8 (0.80)	1302 (0.36)
	3	909 (0.25)	360,910	13,201	68 (7.55)	6598 (1.84)	10 (1.10)	451 (0.13)	8 (0.88)	1506 (0.42)
	4	807 (0.23)	350,660	7537	63 (7.89)	5717 (1.64)	17 (2.11)	337 (0.10)	9 (1.12)	1588 (0.45)
	5	667 (0.21)	322,477	3546	54 (8.24)	4993 (1.56)	13 (1.95)	322 (0.10)	12 (1.80)	1688 (0.52)
	6	481 (0.22)	220,398	3860	25 (5.26)	3292 (1.51)	5 (1.04)	246 (0.11)	6 (1.25)	1324 (0.60)
	7	209 (0.15)	136,522	2123	9 (4.45)	1985 (1.47)	4 (1.91)	145 (0.11)	2 (0.96)	881 (0.65)
	8	92 (0.14)	66,297	493	5 (5.62)	922 (1.40)	1 (1.09)	66 (0.10)	3 (3.26)	456 (0.69)
	9	13 (0.11)	11,312	41	2 (15.4)	193 (1.72)	2 (15.4)	15 (0.13)	0	86 (0.76)
	10	1 (0.04)	2548	1	0	59 (2.34)	0	6 (0.24)	0	23 (0.90)
	Overall	5325 (0.24)	2,189,800	93,906	423 (8.03)	45,873 (2.11)	85 (1.60)	3306 (0.15)	54 (1.01)	10,566 (0.48)

Table 2 shows the at least one cumulative false positive test and true positive probability after 10 visits. The cumulative probabilities of at least one false positive test, false positive referral, and true positive were 18.2, 1.5, and 5.7 %, respectively, after 10 visits. The cumulative probability of first false positive test was 15.9 % (not shown in Table).

**Table 2:** Cumulative probability of at least one false positive (FP) test, FP referral, and true positive after 10 screening visits

Screen number	FP test probability	Cumulative FP test probability	FP referral probability	Cumulative FP referral probability	True-positive probability	Cumulative probability of true-positive
1	0.0407	0.0407	0.0034	0.0034	0.0047	0.0047
2	0.0216	0.0614	0.0014	0.0048	0.0037	0.0084
3	0.0187	0.0790	0.0013	0.0061	0.0042	0.0125
4	0.0167	0.0944	0.0010	0.0071	0.0045	0.0170
5	0.0159	0.1089	0.0010	0.0081	0.0053	0.0222
6	0.0154	0.1226	0.0011	0.0092	0.0060	0.0281
7	0.0149	0.1357	0.0011	0.0103	0.0065	0.0344
8	0.0141	0.1479	0.0010	0.0113	0.0069	0.0411
9	0.0174	0.1627	0.0015	0.0128	0.0076	0.0483
10	0.0224	**0.1822**	0.0024	**0.0151**	0.0090	**0.0569**

Bold numbers indicate the final cumulative number

The cumulative probability of having at least one false positive test was significantly higher in those who had a history of lump compared to those with no history of lump, 45.2 vs. 17.2 % estimated for 10 visits. Cumulative probability of at least one false positive referral and true positive in women who reported any symptoms in screening history were 3.8 and 12.6 %, respectively, compared to 1.4 and 5.3 %, in women with no history of any symptom. (Table 3) There was some increase in the probability of false positive test before the visit with a lump compared to visits with no lump, though true positive probability did not differ (see supplementary table, S1). Women who reported lump or secretion more than one time had higher cumulative probability of at least one false positive test than women who reported lump or secretion once in screening history, 47.8 vs. 44.0 % for lump and 39.8 vs. 33.4 % for secretion, respectively. However, cumulative probability of true positive was lower in women who reported symptoms more than one time compared to one time in screening history.

**Table 3:** Cumulative probability of at least one false positive (FP) test, FP referral, and true positive in women with a history of symptoms

Symptoms history	Cumulative probability of false positive test	Cumulative probability of false positive referral	Cumulative probability of true positive
Lump			
Yes	0.4516	0.0332	0.1630
1 time	0.4401	0.0357	0.2002
>1 time	0.4780	0.0268	0.0650
No	0.1721	0.0146	0.0531
Retraction			
Yes	0.2464	0.0261	0.0903
1 time	0.2662	0.0429	0.1868
>1 time	0.2342	0.0159	0.0368
No	0.1807	0.0204	0.0567
Secretion			
Yes	0.3477	0.0789	0.0638
1 time	0.3339	0.0677	0.0691
>1 time	0.3981	0.1239	0.0489
No	0.1811	0.0146	0.0569
Any symptom			
Yes	0.3843	0.0377	0.1262
1 time	0.3938	0.0422	0.1730
>1 time	0.3694	0.0315	0.0533
No	0.1699	0.0138	0.0530

**Fig. 3:**
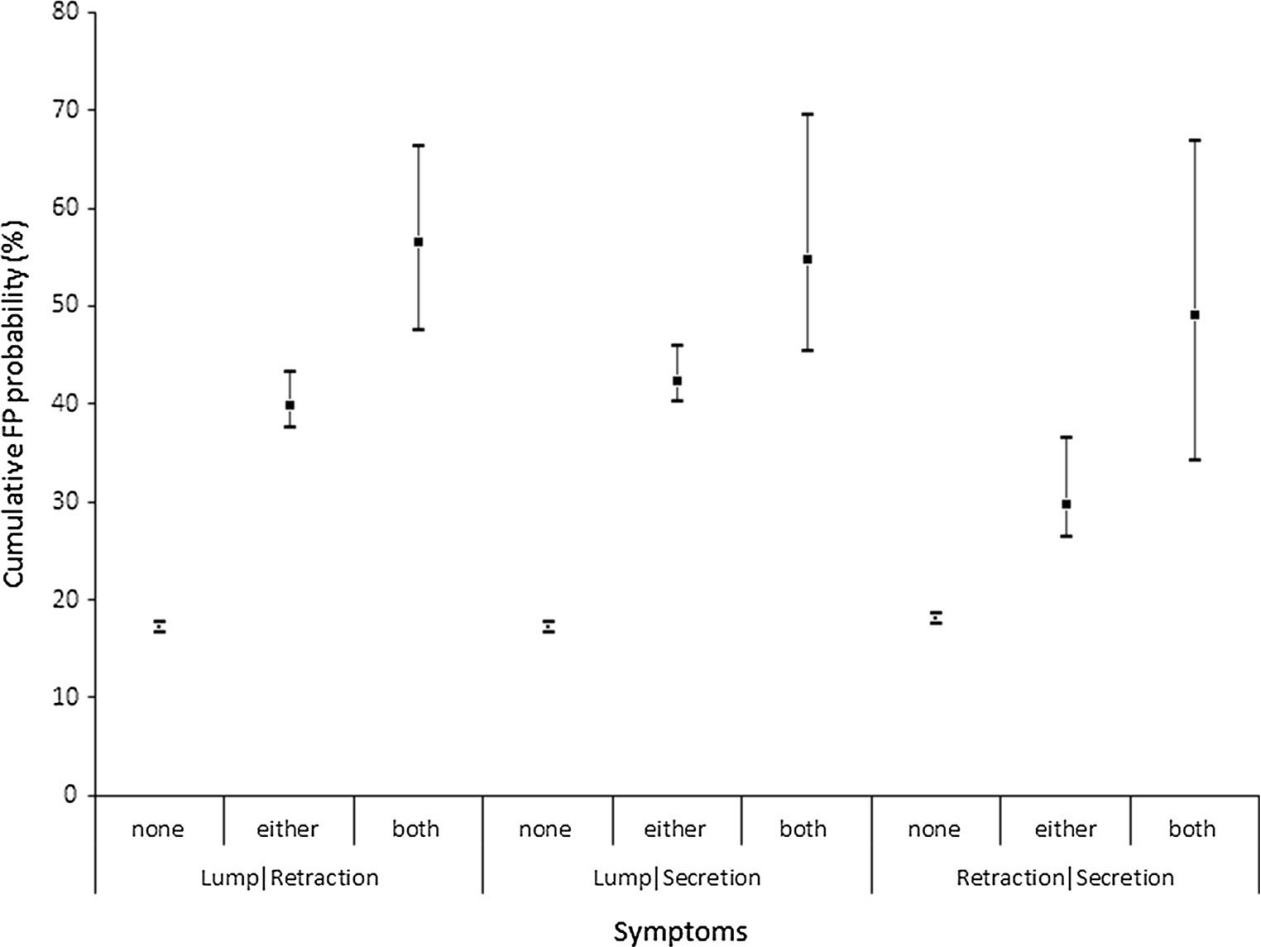
Cumulative probability of at least one false positive (FP) test among women reported symptoms at screen

The cumulative false positive probability in women who reported ‘lump and retraction’ was higher, 56.5 % (95 % CI 47.4–66.3) compared to those who did not report either symptom, 17.1 % (95 % CI 16.6–17.7) (Fig. 3). For Women who reported ‘lump and secretion, the cumulative false positive test probability was 54.8 % (95 % CI 45.3–69.6).

## Discussion

Our study found significantly higher cumulative false positive test and true positive probability among those who reported symptoms at screen compared to those who did not report any symptoms. The cumulative risk of false positive test (after 10 rounds) with any symptom was 38 % and that without was 17 %. Lump was associated with the highest cumulative false positive risk of 45 %, retraction 25 %, and secretion 35 %.

The overall cumulative probability of at least one false positive test was 18 % after 10 screening visits at age 50–69 years and the false positive test probability was 3.6 % at the first visit at age 50–51 years. Our results are consistent or somewhat lower with that of previous studies from other European countries [10–17]. A study from Norway reported a higher cumulative false positive risk (23 %) than the current study [10]. Another study estimated a 21 % cumulative false positive probability projected after 10 screening visits, based on the results of three consecutive screening visits performed in four counties [11]. A retrospective cohort study from Spain projected the cumulative false positive risk to be 20.4 % after 10 screening visits [12]. Cumulative false positive probability from a randomized trial in the UK (2010) was 20.5 % over seven screening rounds [16]. A Danish study [14] made the prediction, based on 3–5 observed screening rounds, of cumulative false positive test probability slightly lower than that of our study. However, the false positive test probability at first screen was higher (5.7 %) in Copenhagen than that of the current study. In the Netherlands, Otten et al. (2013) found lower cumulative false positive risk after 13 consecutive screening examinations than that of our study, but they expected higher estimates after digital mammography was introduced in 2003 [18, 19]. Nonetheless, there were some variations between countries in the methodology and health service system, such as age at first invitation [10, 14, 18], projected estimates based on few observed rounds [11, 18], and lower recall proportion of <1 % at subsequent screens [18, 20] compared to 2.2 % in our study and <3 % in European guidelines [21], while estimating the false positive risk.

Studies conducted in the USA have reported much higher risk of cumulative false positive tests than that of the current study [8, 22–25]. In the US, Breast Cancer Surveillance Consortium (BCSC) data from all women (*n* = 88,455) first screened at age 50-69 years between 1996 and 2010 estimated the cumulative false positive risk to be 41.9 % after eight screens annually or biennially [24]. The reason for lower estimates in our study may be due to different program organizations in Finland than in USA as well as variation in age at first screening, definitions of recall, recording and coding of screening data, screening interval, etc. [26]. Also, the European quality standards [21] are adequately met by the Finnish screening program.

Together with the cumulative probability of ‘at least one’ false positive, this study also estimated the cumulative ‘first’ false positive test and true positive probability. The cumulative first false positive probability was 16 % as we considered only the first false positive mammography result, excluding later false positive findings of the same woman. Hence, the estimate is lower than the ‘at least one’ false positive estimate. Also, the lower probability of false positive in our study may be due to the exclusion of the first visits made at later age, hence removing contamination of newcomers at later visits with prevalent screens. Our study estimated the cumulative true positive probability to be 6 % after 10 screening visits. A study in the Netherlands estimated similar cumulative cancer detection risk after 13 consecutive screening examinations [18]. We are not aware of other studies on cumulative true positive estimates after 10 screening visits.

No prior studies have estimated the cumulative probability based on reported symptoms with a complete follow-up information. Women who reported having symptoms, especially lump and secretion at screening visit, current or at any previous visit, were significantly more likely to have a false positive test and true positive result than women with no symptoms reported. The cumulative false positive probability in women with a history of lump was 45 % compared to 17 % with no lump. When considering the full visit history of women with lump, before and after visit with lump, the higher probability of false positive test before the visit with a lump indicates that there was a possibility that some unspecific changes in the mammograms had been seen even several years before the visit when a lump was reported. On the other hand, after reporting the first symptom there was no increase in the probability of false positive test and true positive results in the later visits. This means that woman was treated and no cancer was detected in later visits. Women were more likely to be true positive if they reported symptoms at screen; cumulative true positive probability of 16 % was compared to 6.5 % with no reported lump. Similarly, women who reported both ‘lump and retraction’ in the same visit had cumulative false positive test probability of 56.5 % (95 % CI 47.4–66.3) compared to 17.1 % (95 % CI 16.6–18.3) without symptoms. Similar results were found in women with other possible combination of symptoms. Taking into account the information on breast symptoms, there is a concern for the radiologist whether or not to recall the symptomatic women. Also, variation in the false positive probability by symptom status, number of times symptom was reported, shows that not all symptoms are equally sensitive. At the same time, the findings also showed benefits of evaluating symptoms information on the performance (more cancers detected) of mammography screening program.

One of the limitations of this study is the missing information on some important risk factors such as hormone use, breast density, and family history of breast cancer, while estimating the cumulative false positive and true positive probability in relation to symptoms. The missing information (1.2 % of total visits) on symptoms was due to incomplete reporting by some centers in the early years of the program. Women recalled but not referred to hospital and women referred but with no cancer in histological confirmation who may have had a cancer before the next screening visit (interval cancer), were not taken into account in this study. Other performance measures of screening program, including interval cancers and mortality as stated by Otten et al. [18] and Tornberg et al. [27], in relation to breast symptoms need to be evaluated thoroughly.

The current study is based on a large nationwide screening cohort with complete follow-up of the women up to maximum 10 visits (21 years). The high participation rate (>85 %) in the screening program and few opportunistic screening means false positive probability estimates over the 10 screens equals the lifetime risk of false positive test in Finland, which is similar to that reported by a Danish study [14]. The radiologists learning of the previous mammography results and the small difference between ‘at least one’ and ‘first’ cumulative probability estimates form the basis to conclude independence between false positive risks at subsequent screen.

In conclusion, the current study showed that information about breast symptoms, especially lump, cause harms in terms of extra false positive findings. The risk varies substantially, depending on symptom types and characteristics. At the same time, more cancers were detected in symptomatic women suggesting benefits of evaluating symptoms information in the program. Information on breast symptoms influences the balance of absolute benefits and harms of screening for the individual woman, and should be considered carefully in breast cancer screening programs.

## Electronic supplementary material

Below is the link to the electronic supplementary material.

Supplementary material 1 (PDF 267 kb)

## Abbreviations

IARC: International Agency for Research on Cancer
FCR: Finnish Cancer Registry
GLM: Generalized linear model
FP: False positive
CI: Confidence interval
UK: United Kingdom
USA: United States of America
BCSC: Breast Cancer Surveillance Consortium
