# Supplementary material for: Cumulative risk of false positive test in relation to breast symptoms in mammography screening: a historical prospective cohort study
Source: Breast Cancer Res Treat. 2016 Aug 5;159(2):305–13. doi: 10.1007/s10549-016-3931-8 (PMC5012157; doi:10.1007/s10549-016-3931-8)
Supplement: Supplementary file 1 — Supplementary material 1 (PDF 267 kb) [file 10549_2016_3931_MOESM1_ESM.pdf]

Title: Cumulative risk of false positive test in relation to breast symptoms in mammography screening: a historical prospective cohort study

Deependra Singh[1,2], Janne Pitkäniemi[1], Nea Malila[1,2], Ahti Anttila[1]

[1]**Finnish Cancer Registry, Helsinki, Finland**

[2]**School of Health Sciences, University of Tampere, Tampere, Finland**

Correspondence to: Mr. Deependra Singh, School of Health Sciences, Medisiinarinkatu 3, FI-33014, University of Tampere, Tampere, Finland.

Tel. no.: +358-449887191; (E-mail: deependra.singh@cancer.fi or nep.singhd@gmail.com)

Supplementary table (S1):
Probability of false-positive (FP) test and true-positive in women before the visit with lump or visit with no history of lump

Screen number	FP test probability with no lump	FP test probability before visit with lump	True-positive probability with no lump	True-positive probability before visit with lump
1	0.0390	0.0630	0.0042	0.0042
2	0.0202	0.0377	0.0033	0.0030
3	0.0177	0.0300	0.0039	0.0046
4	0.0156	0.0326	0.0042	0.0046
5	0.0146	0.0285	0.0049	0.0030
6	0.0142	0.0223	0.0056	0.0063
7	0.0138	0.0379	0.0061	0.0047
8	0.0133	0.0299	0.0066	0
9	0.0164	0	0.0070	0
10	0.0220	0	0.0086	0
